# A Preliminary Study on Hymenal Morphology in Indian Females Without a History of Sexual Intercourse Presenting With Gynaecological Complaints

**DOI:** 10.7759/cureus.79336

**Published:** 2025-02-19

**Authors:** Swapnil Akhade, Pushpawati Thakur, Kiran Akhade, Pankaj S Ghormade, Harshad Bagde, Nilajkumar D Bagde, Vinita Singh, Krishnadutt Chavali

**Affiliations:** 1 Forensic Medicine and Toxicology, All India Institute of Medical Sciences, Raipur, Raipur, IND; 2 Obstetrics and Gynaecology, All India Institute of Medical Sciences, Raipur, Raipur, IND; 3 Epidemiology, Regional Leprocy Training and Research Institute, Raipur, IND; 4 Obstetrics and Gynaecology, All India Institute of Medical Sciences, Nagpur, Nagpur, IND

**Keywords:** colposcopic examination, hymen morphology, non-sexual genital infections, nonspecific findings, physical activities

## Abstract

Forensic examiners need to conduct detailed physical and genital examinations in medicolegal cases to testify as experts. In female genitalia, the hymen is a mucosal tissue surrounding the vagina that varies in appearance and thickness among individuals, adding to the complexity of female genital anatomy. There is limited research on the hymen, but understanding its structure and possible injuries is important for forensic examinations. This study aimed to look at the hymen's structure in female participants without a history of sexual intercourse who came to the gynaecology outpatient department (OPD) for gynaecological complaints. A detailed hymen examination was conducted, along with colposcopic photo documentation. This study also explores how daily physical activities and nonsexual genital infections affect the hymen. The study involved a cohort of 41 female participants aged between 3 and 30 years, with a median age of 17. The annular hymen type was identified as the most frequently observed. Additionally, superficial notches were documented as the predominant non-specific finding in the hymenal membrane, occurring in 58.5% of cases. Erythema was observed on the hymenal membrane during a genital infection; however, the study population showed no additional signs of inflammation associated with the hymen.

## Introduction

The hymen, a thin membrane located at the vaginal entrance, exhibits a range of anatomical variations and nonspecific findings that may be easily overlooked by less experienced examiners. These variations can present as notches, mounds, or partial clefts, which, without proper training, might be mistakenly interpreted as indicators of healed hymenal transection. In addition, these nonspecific findings include ridges and tags and erythema or hypervascularity. The presence of erythema or redness also indicates irritation or inflammation. Given the complexity and variability of hymenal anatomy, registered medical practitioners need to develop a comprehensive understanding of these potential variations. This knowledge is vital to ensure accurate assessments during examinations and avoid misdiagnosis that could have significant implications in forensic cases. Thorough training and awareness of these anatomical subtleties will enhance the reliability of the examination process and the integrity of the findings obtained in forensic contexts. This knowledge enhances their skills and confidence when conducting baseline assessments and documentation, particularly in medicolegal contexts [1-3]. The hymen is the mucosal tissue that encircles the vagina. This membrane can significantly vary in appearance and thickness among individuals, and it typically extends into the lining of the vagina, adding to the overall complexity of the anatomy [4].In the Indian scenario, when female patients visit doctors with any gynaecological complaints, most of the physicians rely more on patient-reported symptoms rather than conducting thorough genital examinations due to heavy workloads, especially in public hospitals. This can impact the credibility of these medical professionals in legal or forensic settings when commenting on normal variations in genital interpretations in a medicolegal setting. In medical schools, during clinical teachings in gynaecology postings, the focus is primarily on managing patient symptoms and clinical signs. During the genital assessment, structures like the hymen often receive inadequate attention, and many practitioners feel uncomfortable examining or discussing them in detail. Nonetheless, professional societies advocate complete genital examinations, and meticulous description should be a routine part of comprehensive physical assessments.

As per Indian law, any registered Medical Practitioners (M.B.B.S) doctors, irrespective of their experience and expertise, can examine survivors of sexual assault [5,6]. While framing opinion in penetrative sexual assault cases, the most significant findings are genital injuries that also include recent tears and scars on the hymen, as described in previous studies conducted on sexually assaulted females [7-10].

Few authors in Indian forensic medicine textbooks often lean towards broad statements that old transections, tears or hymenal scars or changes in the hymen can also result from strenuous physical activity, trauma, scratching, poor hygiene, cycling, dancing, and infections of the genital system [11,12]. Some authors mention that the hymen does not usually rupture when dancing, jumping, riding, or doing vigorous exercise, but they might be caused by scratching or irritation of the hymen due to bad hygiene [13]. In sexual assault cases, defence lawyers in India often refer to these broader terms used in the Indian textbook on Forensic Medicine. They frequently inquire about the potential for injuries to the hymen, as noted in examination reports, to be attributed to non-sexual genital infections or routine physical activities such as cycling, running, dancing, and other sports that involve similar physical activities the survivor is involved in.

Henceforth, studying hymenal morphology and the impact of regular physical activities and sports-related activities are crucial to creating evidence-based data in forensic medicine.

Evidence-based data on the normal anatomy and morphological variations in the hymen has been documented previously, but most existing knowledge regarding hymenal differences originates from studies conducted in Western populations. This study tried to evaluate colposcopic photo documentation of hymenal morphology in an Indian context among female participants presenting with gynaecological complaints. This study investigates normal morphological variations of the hymen while also exploring the potential relationship between signs of inflammation or hymenal injuries and nonsexual genital infections. The study sought to establish a correlation between various physical activities - including cycling, running, dancing, and outdoor sports - and their potential effects on the hymen in females.

## Materials and methods

Objectives

1. To study variations in hymen morphology by colposcopic camera-based examination in female participants with no history of sexual intercourse, sexual abuse or any traumatic genital injuries with bleeding per vaginum.

2. Medical evaluation of hymenal findings in female participants without any history of sexual intercourse or genital injuries reported to Gynaecology OPD for vulvovaginal symptoms like vaginal discharge, irritation, itching, genital infections, menstrual disturbances or any other gynaecological condition.

The type of research used in this study is quantitative with a cross-sectional research design; we conducted a detailed examination of the hymen to see the relationship between physical activities and nonsexual genital infections with the incidence of injuries or any inflammatory changes in hymenal tissue. This research was conducted in the OPD, Department of Obstetrics and Gynaecology, AIIMS Raipur and the Department of Forensic Medicine, AIIMS Raipur. The study was conducted for three years, from 29 September 2020 to 28 September 2023, and all consenting females visited Gynaecology OPD, satisfying the inclusion and exclusion criteria during the study period. A total of 41 cases were included in this study.

Inclusion criteria

All female participants visiting Gynaecology OPD for gynaecological-related complaints such as pelvic pain, vaginal discharge, irregular menses, vaginitis, etc. and requiring genital examination for their medical management were included.

Exclusion criteria

Female participants with a history of sexual intercourse, congenital genital anomalies, vaginal surgery, or penetrating genital injuries were excluded from the study.

Method

We obtained the consent of female participants above 18 years and those below 18 years of age from their parents/legal guardians. The participants were screened for significant behavioural or medical history suggestive of sexual contact or any other conditions mentioned in the exclusion criteria. The patients were asked to complete a pre-validated questionnaire concerning their health history, home, education, and past significant history. A comprehensive local examination was done after written informed consent for a gross visual and colposcopic camera inspection of the hymen, including surrounding tissues of the vulva in Gynaecology OPD. A complete examination was conducted by the female doctor (PW) in the presence of a disinterested female witness. The patients were placed in a lithotomy position, and external genitalia were carefully examined using the separation of labia majora and minora by traction technique. Care was taken to ensure that the identity of the person examined was not revealed in the photograph. The colposcopic hymenal photograph findings were interpreted in the Department of Forensic Medicine. All investigators, including three gynaecologists and three forensic experts, met at least once a week to ensure the consistency of data collection, including locations of notches, mounds, clefts, etc. and injuries, if any, found during examination. Non-specific hymenal findings and hymenal injury (if any) were properly assessed after discussing photo documents of the hymen. We used descriptive terminology from the American Professional Society for Abuse of Children (APSAC) guidelines and an anatomical atlas to define the variables to ensure precise and consistent classification [14, 15].

Data was entered into a Microsoft Excel tool (Microsoft Corp., Redmond, WA, USA), and observational data from the proforma was recorded in the master chart. Subsequently, IBM SPSS version 28.0.1 (IBM Corp., Armonk, NY, USA) was used to analyze the results using various statistical methods and essential filtering tools. The chi-square test was applied to assess correlation.

## Results

**Figure 1:**
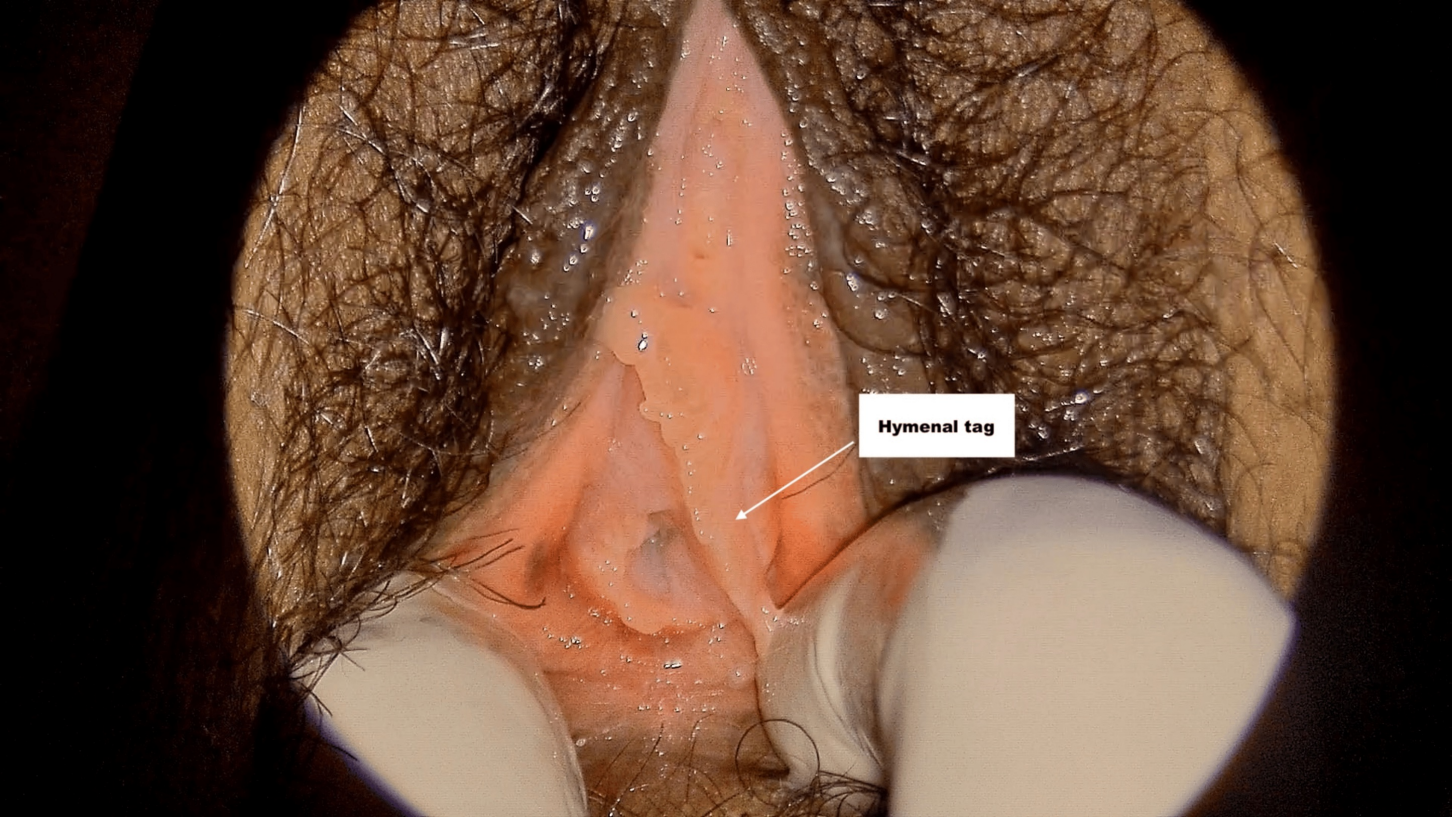
Annular hymen with hymenal tag Annular hymen with a hymenal tag forming a connection between the vestibule and lateral aspect of the hymen.

**Figure 2:**
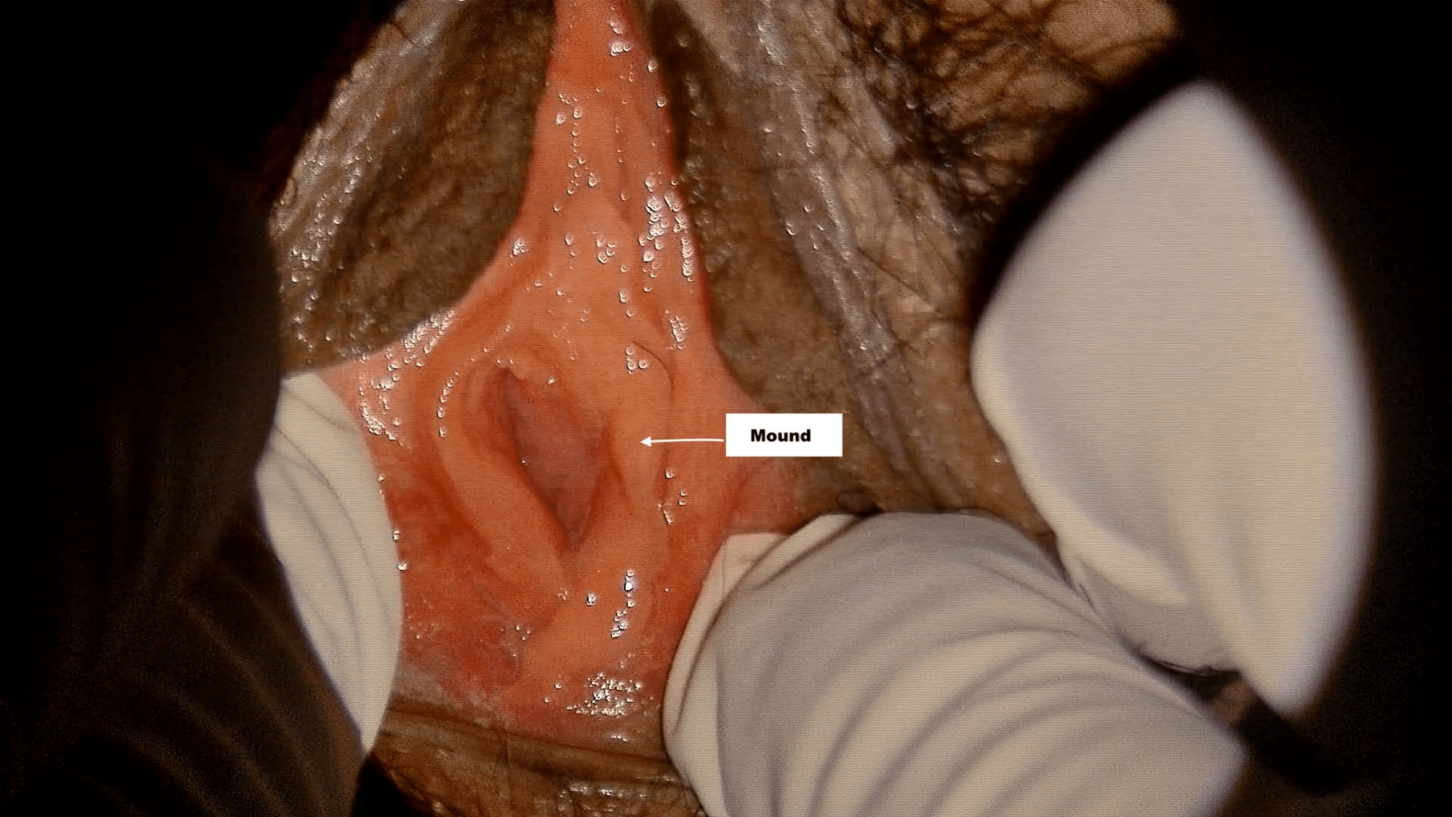
Annular hymen with a mound located at the 3 o'clock position This is an oval-shaped annular hymen with thickened smooth margins and a mound (elevation of mucosa) at the 3 o'clock position.

**Figure 3:**
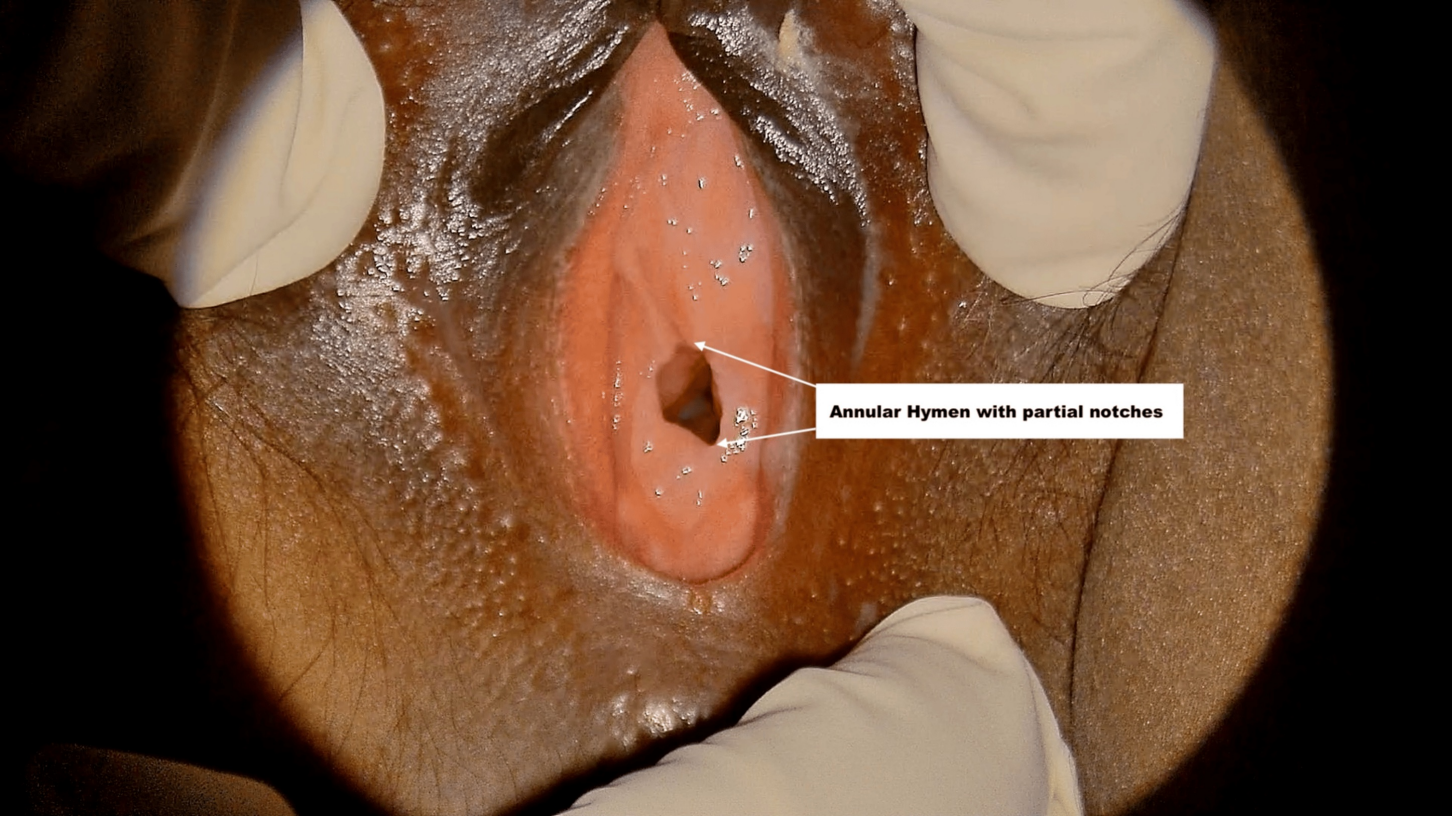
Annular hymen with a superficial notch at 12 o'clock and 5 o'clock position Annular hymen with a superficial notch (<50% of the width of membrane).

**Figure 4:**
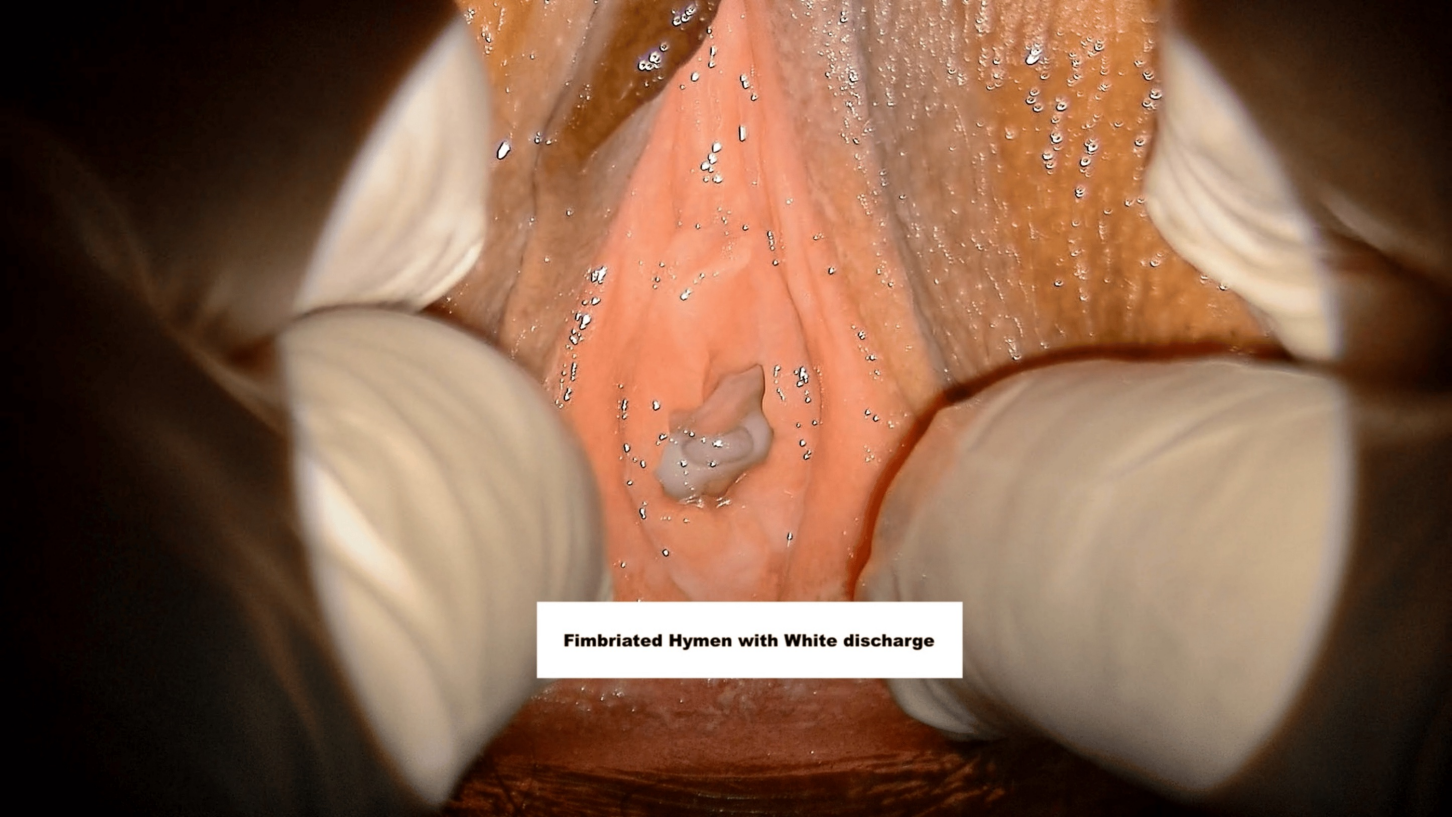
Fimbriated hymen A fimbriated hymen has multiple folds/notches along the hymenal edge.

**Figure 5:**
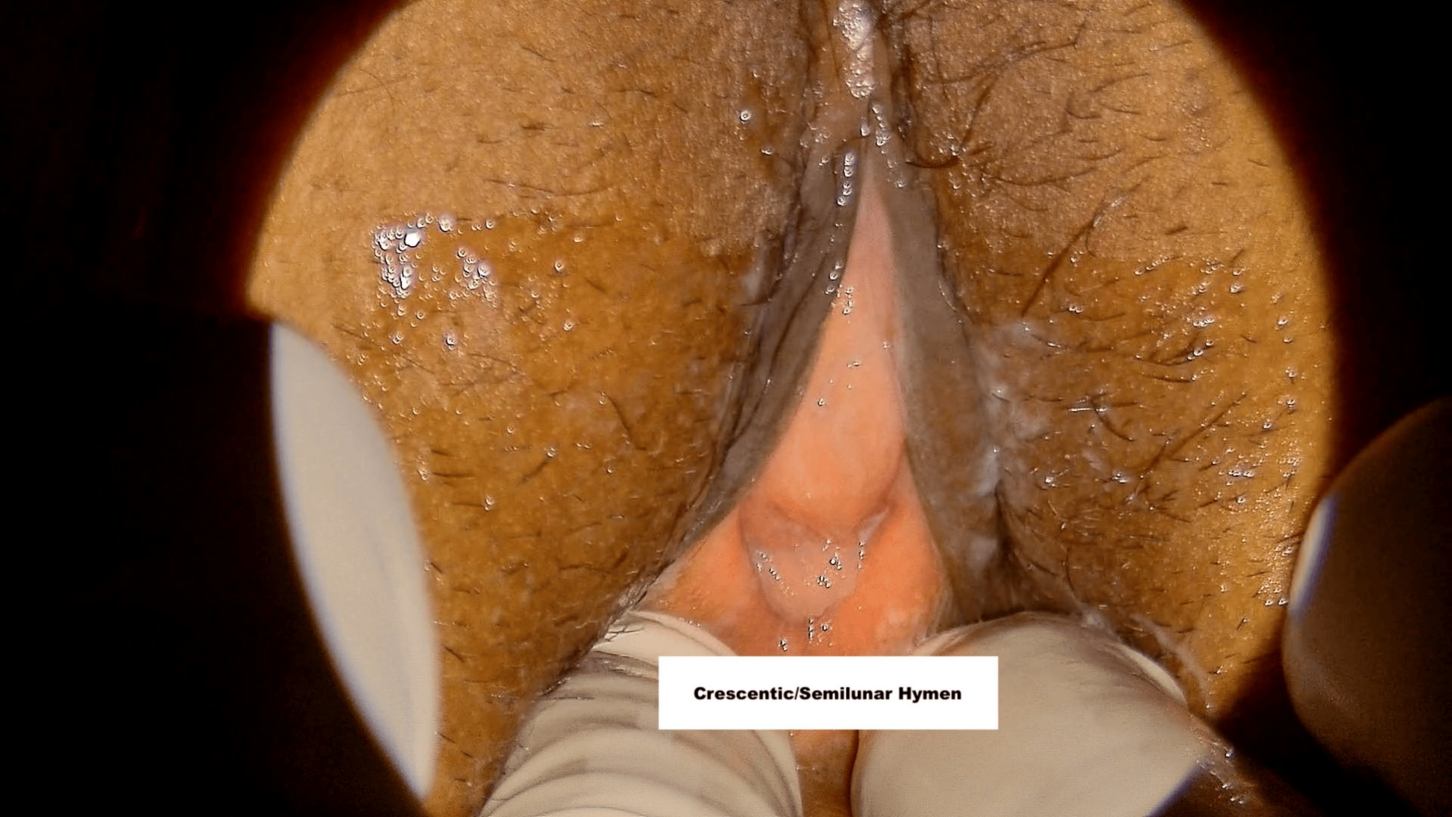
Crescentic/semilunar hymen Crescentic hymen has no definable hymenal tissue between 11 and 1 o'clock.

The hymen’s appearance is characterized by a ring of mucosal tissue that encircles a central opening. This structure can vary significantly, and Figures 1-5 provide a comprehensive overview of the different types of hymens and their related features. Figure 1 illustrates a bump or mound shape. This is a nonspecific finding in hymenal morphology. Figure 2 depicts hymenal tags, which are fleshy protrusions that adhere to the margins of the hymenal membrane. These tags can vary among individuals and may indicate a degree of elasticity or changes in the hymenal structure. Figure 3 showcases a superficial notch located at the 12 o’clock and 6 o’clock positions on the hymen. Figures 1-3 illustrate various nonspecific findings of the annular type of hymen. Figure 4 presents a fimbriated type of hymen characterized by multiple superficial notches along the edge. This type often features a fringed appearance, reflecting a segmented or undulating shape. Figure 5 illustrates a crescentic type of hymen, notable for its crescent shape with a deficiency of tissue present between the 11 o’clock and 1 o’clock positions. This variation can indicate differences in developmental or anatomical factors influencing the hymen’s morphology. Each of these figures highlights the diverse range of hymenal morphology, which can have clinical significance in various contexts, including gynaecological examination in medicolegal cases.

Table 1 presents the operational definitions used to describe various morphologies of the hymen and nonspecific findings according to APSAC guidelines.

**Table 1:** Operational definitions of variations and nonspecific findings in hymen

Hymenal configuration	Defined as crescentic, annular, transitional, fimbriated, tulip, imperforate, microperforated, cribriform and keyhole
Gaping hymenal orifice	Presence of a visible hymenal orifice with the child in supine position and thighs abducted, but without labial separation/traction
External hymenal ridge	A midline longitudinal ridge of tissue on the external surface of the hymen
Peri-hymenal band	Small band of tissue lateral to the hymen that forms a connection between the peri-hymenal structures and the wall of the vestibule
Hymenal bump	A solid elevation of hymenal tissue, which is wider or as wide as it is long, located on the edge of the hymenal membrane
Hymenal tag	An elongated projection of tissue, rising from any location on the hymenal rim part of the hymenal edge folding outwards or inwards
Hymenal notch (Folded hymenal edge) Superficial and Deep	A U- or V-shaped concavity that dips beneath the baseline but not through the entire width of the membrane. Areas appearing as concavities because of proximity to hymenal projections are not included. A superficial notch is smaller than or equal to 50% of the width of the membrane, a deep notch more than 50%.
Hymenal transection	A notch that extends through the entire width of the hymenal membrane

As depicted in Table 2, 41 female participants participated in the study, ranging from 3 to 30 years. The median age was 17 years, and the interquartile range (IQR) was 14 to 22 years. All participants reported to the gynaecology outpatient department without any history of sexual abuse or consensual sexual relations. According to the modified Kuppuswamy scale, 73% of female participants belong to the middle class, while 27% belong to the lower socioeconomic class.

**Table 2:** Sociodemographic characteristics (n=41)

Sr. no.	Characteristics	Number	Percentage
1	Age in years		
	0-6	5	12.20
	7-12	3	7.32
	13-18	13	31.70
	>18	20	48.78
2	Puberty		
	Pre-Pubertal	8	19.51
	Post-Pubertal	33	80.49
3.	Socioeconomic Class		
	Upper Middle (Class II)	14	34.15
	Lower Middle (Class III)	16	39.02
	Upper Lower (Class IV)	11	26.83

Table 3 reveals that superficial notches (58.53%) were the most common nonspecific hymenal findings among the study participants, followed by mounds (12.19%). Deep notches were not found in any cases, while 39.02% did not report nonspecific findings.

**Table 3:** Distribution of nonspecific findings in hymenal morphology (n=41)

Findings	No. of cases	Percentage
No non-specific findings	16	39.02
Superficial notches (< 50%)	24	58.53
Mounds	05	12.19
Peri hymenal band	01	2.44
Hymenal tag	03	7.32

Table 4 shows that among the participants visiting the gynaecology outpatient department (OPD), vaginal candidiasis was the most common diagnosis, followed by abnormal uterine bleeding (AUB). Vaginal infections were reported in individuals aged 4 to 21 years. Hymenal erythema was the only finding noted in one case of vaginal candidiasis and one case of bacterial vaginosis.

**Table 4:** Distribution of diagnosis and inflammatory signs/injury characteristics among study participants PCOD: Polycystic Ovarian Disease; UTI: Urinary Tract Infection

Diagnosis	No. of cases	Erythema		Recent tear		Deep notches or complete transection	
		Present	Absent	Present	Absent	Present	Absent
Abnormal uterine bleeding (Ovulatory)	6	0	6	0	6	0	6
Vaginal candidiasis	14	1	13	0	14	0	14
PCOD	3	0	3	0	3	0	3
UTI	3	0	3	0	3	0	3
Dysmenorrhea	2	0	2	0	2	0	2
Amenorrhea	1	0	1	0	1	0	1
Irregular menses	1	0	1	0	1	0	1
Hypoplastic ovary	1	0	1	0	1	0	1
Endometriosis	1	0	1	0	1	0	1
Endometrial polyp	1	0	1	0	1	0	1
Bacterial vaginosis	1	1	0	0	1	0	1

Table 5 indicates that all participants engaged in low-intensity activities like walking and jogging. Among moderate-intensity activities, running was the most common at 98%, followed by cycling at 39.2% and dancing at 31.7%. Only 12% participated in high-intensity activities such as basketball, football, and weightlifting. There were no reported straddle injuries, genital trauma, or vaginal bleeding, and gynaecological examinations showed no signs of injury or inflammation to the hymenal membrane from these physical activities.

**Table 5:** Distribution of intensity of physical activity among participants.

Intensity	Physical Activity	Numbers	Percentage
Low	Walking, Jogging	41	100%
Moderate	Running	38	92.68%
	Intense Cycling	16	39.02%
	Dancing	13	31.7%
High	Basketball	02	4.87%
	Football	01	2.43%
	Weight lifting	01	2.43%

## Discussion

Most nonspecific findings in hymenal morphology have been reported in the literature and previous studies of nonabused girls [1,2,4,14,15]. Along with the study on normal morphology of the hymen, our study also included the relationship of physical activities, sports participation and nonsexual genital infections to hymenal anatomy.

Few research articles noted variations in hymenal morphology within normal genital anatomy, highlighting a wide range of nonspecific findings. They also framed terminologies for various nonspecific findings, such as peri-urethral/perihymenal bands, longitudinal intravaginal ridges, tags, bumps, vascular changes, labial adhesions, erythema, or even vaginal opening diameter [2,4,16,17]. In our study, we found four nonspecific findings, i.e., superficial notch, mounds, perihymenal band and hymenal tag. Partial notch was the most common nonspecific variation noted in 58.53% of cases. In 2002, a similar study by Heger et al. [4] reported that the perihymenal band was the most commonly observed nonspecific finding, followed by ventral superficial hymenal notches. Their study did not find a complete cleft on the posterior aspect of the hymen in their research. Our findings align with theirs; we found that partial notches were the most prevalent findings and did not observe a complete posterior hymenal cleft among non-abuse females in our study group. In a longitudinal study involving 93 female participants aged between 3 and 9 years, Berenson and Grady found that the most common observation was the presence of a periurethral band. This was followed by at least one intravaginal ridge that extends up to the hymen [18]. They mention that over a period of time, new superficial notches on lateral positions were often found after an age gap of six years, but they did not observe a deep notch (more than half of the membrane) or transection [18]. Though our study is cross-sectional, our findings are coherent with their study.

This is consistent with similar studies conducted by Berenson et al. [19] and Emans et al. [20]. Our study also did not find a deep notch or complete transection at any point in time, and henceforth, we also support the assumptions made by previous studies that the presence of deep notches or complete transections in the lower half of hymen along with the history of trauma or abuse could be considered as an indication.

Our study included participants with no history of penetrating trauma to the genitals who engaged in varying levels of physical activity: low-intensity (walking, jogging), moderate-intensity (running, cycling, dancing), and high-intensity (basketball, football, weightlifting). We found no signs of recent or old injuries to the hymen. These findings align with Emans et al., who reported no direct link between sports activities and hymenal injuries. Emans et al. observed that complete clefts were commonly found in subjects who reported consensual intercourse, whereas they were rarely noted in groups that were not sexually active [20]. Our results show no complete transections on either aspect of the hymen. Similarly, the study conducted by Berenson et al. [19] also did not find complete transection or cleft in the posterior aspect of the hymen, and these findings are consistent with our study. Candida albicans is the most common fungal infection in prepubescent girls [21,22]. Over the vulva, a fungal rash usually appears with raised, well-delineated borders. Although there was a thick, white discharge, no recent hymenal injury or old hymenal tear in the form of complete transection was found in the lower hymen [21]. Among 14 of our study cases diagnosed with vaginal infections, symptomatic vaginal candidiasis was found in 13 cases, and bacterial vaginosis in only one case. Our study did not find any recent hymenal injuries in any fungal infection cases. In our study, we observed erythematous changes in hymen in one case of bacterial vaginosis. However, no recent or old hymen tears or transections were observed among these patients. The most common gynaecological condition among children is vulvovaginitis, and its commonest sign is vulvar redness followed by vaginal discharge [23]. We also found similar findings in our study, i.e., hymenal erythema and itching in two cases of vulvovaginitis. However, previous studies have mentioned that erythema of the hymen is a normal variation and can be found in other gynaecological infections in females [24].

Recommendation

Quality photo documentation by the colposcopic camera should be used to better interpret hymenal findings in medicolegal settings. Understanding normal hymenal variations and nonspecific findings and consistently applying these findings in a medical context is essential for correctly interpreting hymenal membrane findings while examining the genitalia in medicolegal cases.

Limitation of study

Our study had 41 participants. Further research with a more extensive study group is needed to explore the implications of nonsexual genital infections and sports activities on the hymenal membrane.

## Conclusions

The study concluded that hymenal erythema was the only finding noticed due to scratching and nonsexual infections of the genital system; also, in sports activities with no direct penetrating trauma to the genitals, there was no evidence of a recent hymenal tear. Our study did not find a complete cleft/transection extending to the hymenal margin. In this context, hymenal injuries are less common due to routine nonsexual genital infections or physical activities contrary to the concept which is more prevalent among the examiners. The presence of partial clefts and notches, concavities, rim narrowing and thickening are normal variations of hymen found among Indian females.
